# Rapid Genomic Evolution Drives the Diversification of Male Reproductive Genes in Dung Beetles

**DOI:** 10.1093/gbe/evab172

**Published:** 2021-07-28

**Authors:** Mrinalini, Cho Yeow Koh, Nalini Puniamoorthy

**Affiliations:** 1 Department of Biological Sciences, National University of Singapore, Singapore; 2 Department of Medicine, Yong Loo Lin School of Medicine, National University of Singapore, Singapore

**Keywords:** phenotypic traits, transcriptomics, genomics, reproduction, sexual selection

## Abstract

The molecular basis for the evolution of novel phenotypes is a central question in evolutionary biology. In recent years, dung beetles have emerged as models for novel trait evolution as they possess distinct precopulatory traits such as sexually dimorphic horns on their head and thorax. Here, we use functional and evolutionary genomics to investigate the origins and the evolution of postcopulatory reproductive traits in male dung beetles. Male ejaculates that underlie postcopulatory sexual selection are excellent candidates to study novel trait evolution as they are complex, fast evolving, and often highly divergent in insects. We assemble de novo transcriptomes of male accessory glands and testes of a widespread dung beetle, *Catharsius molossus*, and we perform an evolutionary analysis of closely and distantly related insect genomes. Our results show there is rapid innovation at the genomic level even among closely related dung beetles. Genomic expansion and contraction drive the divergence of male reproductive traits and their functions. The birth of scores of completely novel reproductive genes is reinforced by the recruitment of these genes for high expression in male reproductive tissues, especially in the accessory glands. We find that male accessory glands of *C. molossus* are specialized for secretory function and express female, egg, and embryo-related genes as well as serine protease inhibitors, whilst the testes are specialized for spermatogenesis and sperm function. Finally, we touch upon putative functions of these evolutionary novelties using structure-function analysis as these proteins bear no homology to any other known proteins.

Significance

The molecular underpinnings of how novel traits evolve is a central question in biology. Here we use male ejaculates of dung beetles to study the molecular basis of novel trait evolution because ejaculates of male insects are complex and fast evolving. We find the birth of scores of completely novel genes in the genomes of closely related dung beetles, and the reproductive functions of these novel genes are reinforced by their recruitment for high expression in male reproductive tissues. Our study suggests that rapid genomic innovations and genome expansion and contraction drive the evolution and divergence of novel male traits in dung beetles.

## Introduction

Insects are the most diverse among animal groups, and they display a myriad of phenotypes and adaptations that can be attributed to expansion and contraction of their genomes and gene repertoires (Nygaard et al. 2011; Li et al. 2019). Insect gene family expansion and contraction has been the research focus for several well-defined physiological genes and traits that are implicated in immunity, chemoreception, vision, feeding and digestion, detoxification, parasitism, etc. (Holt et al. 2002; Wurm et al. 2011; Chen et al. 2016; McKenna et al. 2016; Papanicolaou et al. 2016; Martinson et al. 2017). Gene duplication, horizontal gene transfers (HGTs) of genomic material from viruses and bacteria, and fast molecular evolution are the some of the mechanisms by which insect genomes evolve (Nygaard et al. 2011; Wurm et al. 2011; Chen et al. 2016; Martinson et al. 2016). Surprisingly however, relatively little is known about the genetic and functional aspects of male reproductive genes or how they evolve in insects, with the exception of well-studied fly models such as *Drosophila melanogaster* (Wigby et al. 2020) or *Aedes aegypti* (Ethan et al. 2019). This is despite the fact that since early 1980s, evolutionary biology has experienced a surge in research on postcopulatory determinants of male reproductive fitness, namely, sperm competition (Parker 1970; Smith 1984). Numerous studies across a broad range of taxa implicate sperm competition as a driving force behind the extraordinary diversity in male and female genital morphology, sperm form, seminal plasma biochemistry, as well as in sex-specific physiology and/or behaviours that can impact competitive fertilization success (Birkhead and Møller 1998; Gasparini et al. 2020; Lüpold et al. 2020; Simmons and Wedell 2020). Females of most insect species not only mate multiply, but they also possess specialized organs for long-term sperm storage, facilitating co-occurrence of sperm from two or more males in the female reproductive tract (Moschilla et al. 2020; Simmons et al. 2020; Wylde et al. 2020). Hence the internal female reproductive environment acts as the competitive arena for fertilization, and much of postcopulatory sexual selection is in fact shaped by variation in male ejaculate and female interactions that can facilitate rapid diversification (Yamane et al. 2015; Lüpold et al. 2016). Therefore, it is necessary to investigate the genes and the gene products specific to male reproductive tissues in order to understand the diversity of male reproductive traits, their origins and evolution, and their role in postcopulatory sexual selection.

However, such studies can be challenging for many reasons, not least of which is the small size of insects and their even smaller reproductive organs, such as the male accessory glands and the testes. There are added difficulties in the case of non-model insects where access to fresh samples and genetic data is often quite limited. For instance, beetles represent the most species-rich taxonomic order among insects, but genomic and functional genomic databases that facilitate genetic and evolutionarily studies are still very scarce in this order (Sayadi et al. 2016). Even in well-studied insect species, evolutionarily meaningful analysis on rapidly evolving reproductive genes is often confounded by the absence of and/or difficulties in identifying homologs and ancestral genes in related species. Furthermore, the functional aspects of these genes are not easily resolved and hence remain largely unknown (Parthasarathy et al. 2009; Bayram et al. 2019). Traditional protein analysis has been replaced by high-throughput proteomics analysis of ejaculates and extracts from male beetles (Peferoen and De loof 1984; Parthasarathy et al. 2009; Goenaga et al. 2015; Gorshkov et al. 2015; Wei et al. 2015; Yamane et al. 2015). However, species-specific sequence databases are still very rarely used in conjunction with protein and proteomics analysis, although this is quite feasible in laboratory cultured insects (Bayram et al. 2017; Bayram et al. 2019). Recently, high-throughput RNA sequencing including microarrays and de novo transcriptomics have been useful for characterization and screening of reproductive genes in flies and in beetles (Parthasarathy et al. 2009; Sayadi et al. 2016; Bayram et al. 2017; Vedelek et al. 2018; Bayram et al. 2019). Our current understanding of the diversity of male reproductive genes and proteins in beetles is therefore limited to a few select species that are of agricultural relevance, such as the rice-flour beetle (*Tribolium castaneum*), the Colorado potato beetle (*Leptinotarsa decemlineata*), and the seed beetle (*Callosobruchus maculatus*) (Goenaga et al. 2015; Yamane et al. 2015; Bayram et al. 2017; Bayram et al. 2019). Considering that beetle biodiversity comprises hundreds of thousands of species, the number of such existing studies is rather sparse and thus far, no dung beetle species have been investigated in detail.

**Fig. 1.**
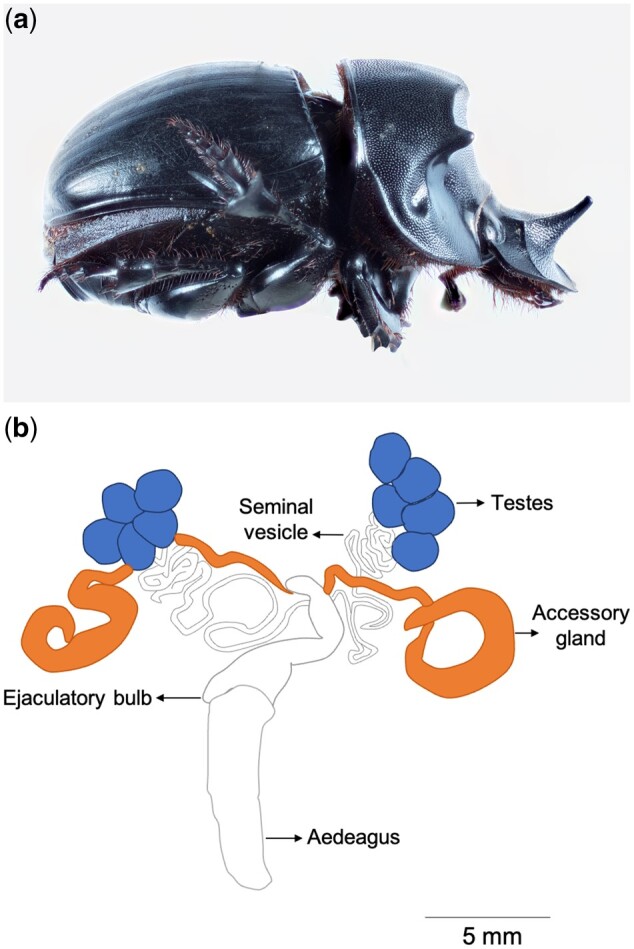
A male *C. molossus* dung beetle and its reproductive system. (*a*) The male can be identified by the presence of a distinctive horn on its heads; and (*b*) schematic showing *C. molossus* male reproductive system, including testes, accessory glands, and a horn-like structure aedeagus used for sperm and ejaculate delivery during mating.

Our study examines the molecular basis for the evolution of novel male reproductive genes in closely related dung beetles. Dung beetles have emerged as models for novel trait evolution as they possess distinct precopulatory traits such as sexually dimorphic horns on their head and thorax (fig. 1*a*). They play an important but largely unseen role in the ecosystem, providing nutrient cycling, soil aeration, and secondary seed dispersal, and are often used as indicators of forest health because they respond rapidly to habitat degradation. Here, we use the widespread and charismatic dung beetle species *Catharsiusmolossus* that is found across South East Asia (fig. 1*a*). The genus *Catharsius* consists of large nocturnal tunnelers and they are sexually dimorphic, with males displaying facial and thoracic horns (fig. 1*a*). Currently there is no information on the ejaculate compositions of *Catharsius*, therefore we first characterize the male genetic profiles from the accessory glands and testes of *C.molossus* (fig. 1*b*), and we then reconstruct the evolutionary histories of these genes. Specifically, we: 1) characterize male ejaculate genes in *C. molossus* using de novo transcriptome sequencing of testes and the accessory glands; 2) investigate the molecular basis of novel reproductive trait evolution in dung beetles by tracing the origins and evolutionary histories *C. molossus* reproductive genes in other insect species; and 3) examine the functional properties of these genes, including expression levels, secretory nature, and structure-function predictions. We find that there is rapid evolution at the genomic level and that expansion and contraction of genomes drive the evolution and diversification of novel reproductive traits even in closely related dung beetles. We find that the accessory glands of *C. molossus* are highly specialized for secretory function and are a particularly important site for the recruitment, the evolution, and the diversification of novel reproductive genes. In contrast, the testes of *C. molossus* are dominated by gene sets that are largely evolutionarily conserved.

## Results

### Transcriptome Sequencing

Total RNA for all tissues ranged between 424 and 770 ng/µl as measured by Bioanalyzer, except for accessory glands from Pulau Ubin for which it was relatively lower at 62 ng/ul (supplementary table S1, Supplementary Material online). RNA quality, as assessed by RNA Integrity Number (RIN) was >7, for all samples. cDNA library construction provided fragment sizes ranging from 600 to 800 bp. The libraries were sequenced on NovaSeq 6000 to a depth of 27.2 and 24.7 million reads for accessory glands and a depth of 20.6 and 27.1 million reads for testes, from Pulau Ubin and mainland Singapore, respectively (supplementary table S1, Supplementary Material online). After removal of contaminant reads, from worm, bacteria, viruses, and mammals, followed by base quality filtering, accessory gland transcriptomes comprised 24.7 and 21.8 million reads and testes transcriptomes contained 18.8 and 24.3 million reads, for Pulau Ubin and mainland Singapore samples, respectively (supplementary table S1, Supplementary Material online). GC content was 41% and 42% for accessory glands from Pulau Ubin and mainland Singapore respectively, and 43% for testes from both localities.

### De Novo Transcriptome Assembly

A total of 310,072 and 222,129 sequences were de novo assembled for Pulau Ubin and mainland Singapore samples, respectively. After filtering for open-reading frames (ORFs), for the presence of start codons, duplicate sequences, gene expression value of at least 1 transcript per million (TPM), and the retention of sequences ⩾200 bp in length, the Pulau Ubin transcriptome assembly consisted of 41,362 sequences with 40 million bases and the mainland Singapore assembly consisted of 37,421 sequences with 39 million bases (supplementary table S1, Supplementary Material online). BLASTP of translated nucleotide sequences with an *e-value* cut-off of 1e-5 returned 31,071 and 27,876 hits against NCBI’s *nr* database for Pulau Ubin and mainland Singapore, respectively.

Following manual curation of the top 100 highest expressed sequences and removal of chimeric assemblies, rRNA sequences, residual contaminant contigs (such as *Escherichia coli*), 45 high-quality and highly expressed sequences were retained for downstream evolutionary and functional analyses for both the accessory glands and the testes (supplementary tables S3 and S4, Supplementary Material online). We found that both the gene sets were tissue specific, i.e., none of the 45 highest expressed genes from one tissue was shared with the other tissue. Therefore, we henceforth designate the gene sets as *accessory gland genes and testes genes* for ease of reference in all subsequent sections.

### Evolutionary Analysis

**Fig. 2:**
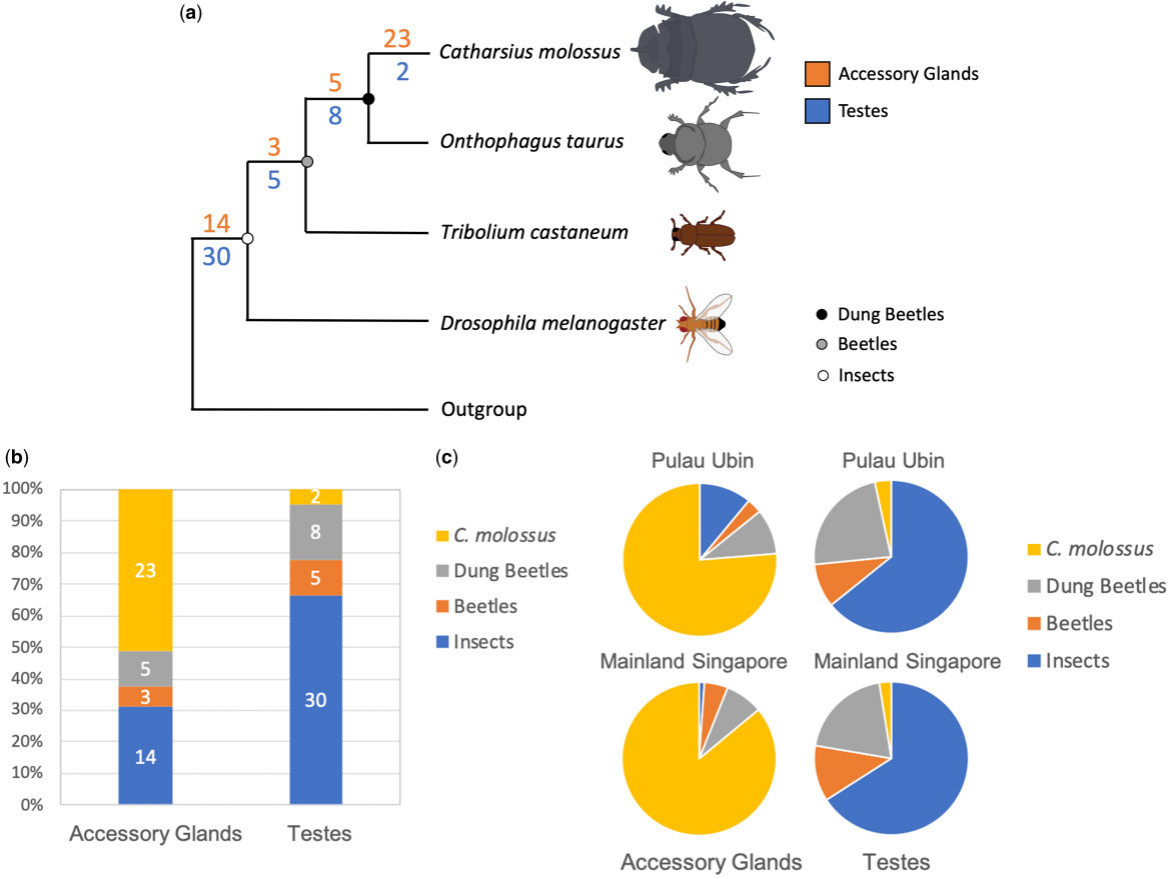
Rapid evolution of novel reproductive genes in the dung beetle, *C. molossus.* (*a*) The evolution of 45 highest expressed genes, derived from de novo transcriptomics of *C. molossus* reproductive tissues, is plotted on a schematic insect phylogeny. The number of novel *C. molossus* accessory gland genes (in orange) and novel testes genes (in blue) that evolved at each phylogenetic node are shown (black - dung beetles, gray - beetles, and white - insects). Dung beetle accessory glands are a prime site for rapid evolution of novel reproductive genes, with 23 of 45 genes unique to the *C. molossus* genome. Strikingly, only five genes are shared with *O. taurus*, a closely related dung beetle from *Scarabaeinae*, the same subfamily as *C. molossus.* In contrast, a majority of testes genes (30) are highly conserved as far as the fly group (represented by *D. melanogaster*). Eight testes genes evolved in the dung beetle clade whereas only two testes genes were found to be unique to *C. molossus.* (*b*) Proportion of genes in each insect group represented as a bar chart. Over 50% (23) of the accessory gland genes are novel to *C. molossus.* In exactly the opposite trend, 65% (30) of testes genes are conserved in all four insects whereas only 2 genes are novel to *C. molossus*. (*c*) Proportion of gene expression in each insect group. Novel *C. molossus* genes account for over 75% of gene expression in the accessory glands, whereas conserved genes account for ca. 65% of expression in the testes.

We reconstructed the evolutionary histories of the 45 highest expressed genes in *C.molossus* the accessory glands and testes. We searched for these genes in the genomes and protein databases of three insect species: *Onthophagus taurus,* also a dung beetle from the same sub-family as *C. molossus*, *T.castaneum*, a well-studied beetle model species from the same order as *C. molossus,* and the more distant fly group representative, *D.melanogaster.* Our results show that 23 out of 45 accessory gland genes have uniquely evolved in genome of *C. molossus* (fig. 2). Even with the closely related dung beetle *O. taurus, C. molossus* shares only five genes, and the remaining genes are completely absent from the *O. taurus* genome. Only three accessory gland genes are found to be conserved in the genome of the more ancestral, red flour beetle *T. castaneum.* Finally, of the 45 accessory gland genes, 14 genes are found to be shared up to the fly group (*D. melanogaster*).

We found one *C. molossus* accessory gland gene with BLASTp hit to fungi (supplementary table S3, Supplementary Material online, C126_AG_Sequence_19). For this gene, the possibility of HGT from fungi was investigated. Ortholog search and filtering of sequences from EggNOG v.5.0 (Huerta-Cepas et al. 2019) resulted in 25 putative orthologous sequences from different organisms. Among them were several families of fungi and insects, including flies, butterflies, and a mosquito species. Bayesian phylogenetic analysis and 50% majority rule consensus tree re-constructed from protein sequence alignment, rooted using the most distant fungal ancestor *Phytophthera infestans*, showed *C. molossus* on a separate branch strongly supported by a posterior probability of 100% (supplementary fig. S1, Supplementary Material online). All other species formed a single cluster and there was no distinct clustering of either the insect group or the fungal group, with fungal species from different genera interspersed among insect species.

###  

#### Secretory and Non-secretory Gene Composition, Evolution, and Expression

**Fig. 3:**
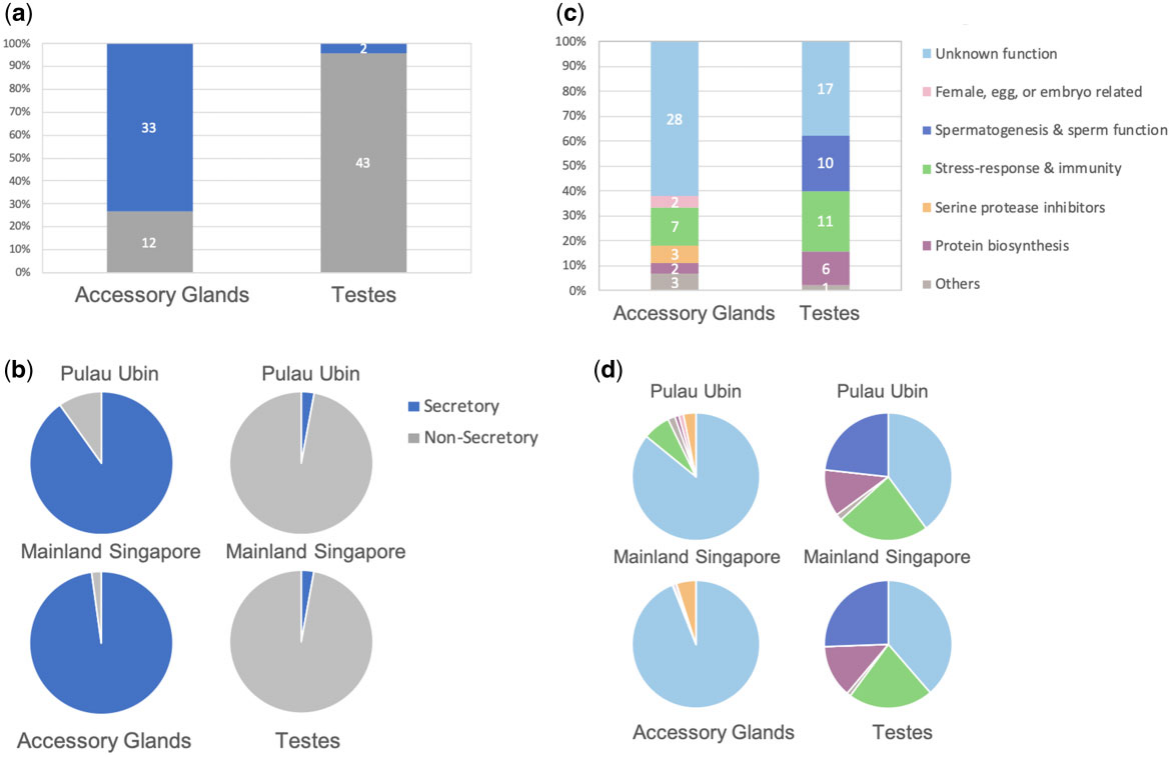
Functional characteristics and gene expression patterns in the reproductive tissues of *C. molossus.* Secretory and non-secretory gene composition (*a*) and gene expression (*b*) in the accessory glands and testes of *C. molossus.* The accessory gland is specialized for secretory function, with 73% (33) of genes being secretory in nature. Secretory genes also account for a majority of gene expression in the accessory glands. In contrast, the testes gene set is composed of mainly non-secretory genes 96% (43) which also dominate expression in the testes at 97%. (*c*) Proportion of genes in each functional category. Both accessory glands and testes are dominated by several genes of unknown function. Stress response and immunity and protein biosynthesis genes are shared across both tissues. The testes contain a set of 10 genes involved in spermatogenesis and sperm function, whereas the accessory glands contain serine protease inhibitors and genes involved in female, egg, or embryogenesis interactions. (*d*) Proportion of gene expression in each functional category. In both tissues, a majority of expression is by genes of unknown function. In the testes, spermatogenesis and sperm function is the second highest expressed category, followed by stress response and immunity and protein biosynthesis genes.

Among the 45 accessory gland genes analyzed, we find that 33 contain a signal peptide in their protein sequence, i.e., they are secretory in function (fig. 3*a*). Of the 33 secretory genes, 22 are novel to the *C. molossus* genome with no functional homologs in other insects. Five secretory genes are conserved up to *O. taurus*, and three are found to be conserved as far as beetles and insects*.* Accessory glands express 12 non-secretory genes of which 11 are conserved in all insect species, whereas one appears to have evolved as a novel gene in *C. molossus*. Secretory genes are over represented in the accessory gland gene set, and moreover they are among the highest expressed genes, accounting for over 80% of gene expression in the accessory glands (fig. 3*b*). In contrast, the testes gene set is dominated by non-secretory genes both in number and in expression (fig. 3*a and b*). Among 45 testes genes, 43 perform non-secretory function, and 30 of them are conserved across beetles and flies. Furthermore, 97% of expression in the testes are from genes that are non-secretory in nature (fig. 3*b*). Only two testes genes contain a secretory signal in their protein sequence, and one each is found to be conserved in beetle and dung beetle clades.

#### Conservation and Expression of Genes in Each Insect Group

In analysis of association between evolutionary conservation status and expression of genes, *Chi-squared* test was not significant for both tissues using a *Pvalue* cut-off of 0.05 (supplementary table S2, Supplementary Material online). In the accessory glands, there is no association between high gene expression and genes newly recruited into the *C. molossus* genome, either in Pulau Ubin or in mainland Singapore samples (*Pvalue* 0.22 for both localities). We also find no association between high gene expression and conserved testes genes in both localities (*Pvalue* 0.22).

### Functional Categories of Genes and Their Expression

**Fig. 4.**
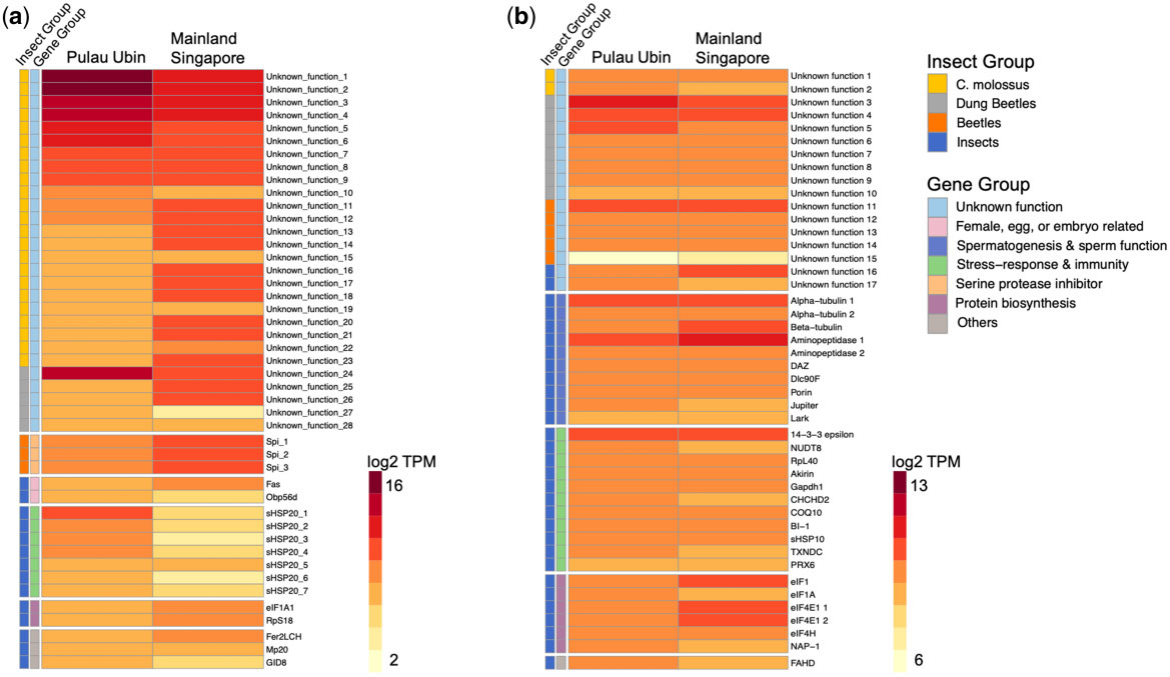
Heatmap of gene expression in the reproductive tissues of *C. molossus.* Log_2_ TPM values are plotted, and genes are grouped by evolutionary and functional categories represented by color-coded bars to the left. (*a*) Accessory glands**:** Genes novel to *C. molossus* and to dung beetles are among the highest expressed in the accessory glands. (*b*) Testes: The expression pattern of genes with unknown function is less striking and are moderate to low. Similar expression patterns are seen in evolutionarily conserved categories, including spermatogenesis and sperm function, protein biosynthesis, and stress response and immunity.

We classified genes into seven broad categories based on their function: (i) unknown function; (ii) female, egg, or embryo related; (iii) spermatogenesis and sperm function; (iv) stress response and immunity; (v) *serine protease inhibitors* (*Spi*); (vi) protein biosynthesis; and (vii) others, containing genes that do not belong to any of the first six categories (figs. 3*c*, 3*d*, and 4). A large proportion of accessory gland genes that are novel to *C. molossus* genome are also among the most highly expressed in both Pulau Ubin and mainland Singapore samples (fig. 3*c*). Some accessory gland genes shared only with *O.taurus,* and therefore specific to the dung beetle clade, have moderate to low expression. We find three moderately expressed *Spi* genes that are beetle clade specific, and seven low expressed *small heat shock protein 20s* (*sHSP20s*) conserved across insects (figs. 3*c*, 3*d*, and 4).

In the testes, functionally annotated and highest expressed genes are also conserved in the genomes of all insects we examined (fig. 3*c and d*). These genes are involved in spermatogenesis and ejaculate-female interactions and likely functionally highly conserved (fig. 3*d*). The third highest expressed category in the testes is a set of eight genes novel to the dung beetle clade, although their functions are currently unknown. Two testes genes that are novel to *C. molossus* genome have moderate expression. In both tissues, we find genes from the “others” category that are involved in an assortment of functions, including protein synthesis, stress response, cell death and apoptosis, immune functions, and energy metabolism.

### Structure-Function Analysis

BLAST search did not return significant hits for some *C. molossus* proteins. Therefore, we used a three-dimensional-structure-based search approach to derive insights into possible functions of these novel *C. molossus* proteins. Sequences for the top five highest expressed genes in the accessory glands and in the testes that had unknown function were submitted to I-TASSER server for structure and function predictions. Our results from I-TASSER suite (Yang et al. 2015) gave relatively low confidence-scores (*C*-scores) for predicted three-dimensional structure, ranging between −3.12 and −1.62 for all five novel accessory gland proteins, and between −3.95 and −1.12 for all five testes proteins (on a scale of −5 to 2) which is consistent with structure predictions of proteins without significant sequence homology to other proteins. However, structural alignment of the top I-TASSER model of novel accessory gland and testes proteins using TM align returned high scores in many cases, indicating a high level of confidence in structural similarity. TM scores (on a scale of 0 to 1, 1 being perfect match) ranged between 0.606 to 0.79 for the accessory glands and 0.524 and 0.961 for testes (supplementary table S5, Supplementary Material online). The PDB structural analogs identified by TM align and their associated UniprotKB protein accession numbers are also shown in supplementary table S5, Supplementary Material online.

To ensure reliability of the predictions, we use the Cscore^GO^ for consensus GO terms associated with each query (including molecular function, biological processes, and cellular components). Cscore^GO^ is the composite of TM scores, root-mean-square deviations of aligned structures, percentage of identity residues, and coverage of aligned part of the structures. The overall Cscore^GO^ for accessory gland proteins ranging between 0.22 and 0.50 on a scale of 0 to 1, with higher score indicating higher confidence in predictions (supplementary table S6, Supplementary Material online). For testes proteins, the overall Cscore^GO^ ranged between 0.07 and 0.61 (supplementary table S6, Supplementary Material online). Further structure-based searches using COFACTOR and COACH (prediction by ligand-binding site) returned weak predictions with ligand-binding C-scores ranging from 0.02 to 0.12 for accessory gland and 0.02 to 0.15 for testes, and enzyme active sites Cscore^EC^ ranging from 0.097 to 0.239 for accessory glands and 0.066 to 0.145 for testes (not shown). Both ligand-binding C-score and Cscore^EC^ are evaluated on a scale of 0 to 1, where a higher score means a more reliable prediction.

## Discussion

### Rapid Evolution of Male Reproductive Gene Novelties in Dung Beetles

We used de novo transcriptomics to characterize gene composition and gene expression in the male reproductive tissues of *C. molossus.* We defined rapid evolution as the rate of acquisition and loss of de novo protein-coding genes in a species, and we traced the evolutionary histories of *C. molossus* reproductive genes in the genomes of three other insects, a closely related dung beetle *O. taurus*, the model beetle species, *T. castaneum*, and the distant fly model *D. melanogaster*. We find that both testes and accessory glands of *C. molossus* express a complex array of genes that are unique to each tissue, i.e., none of the genes in one tissue is shared with the other tissue, and there is no overlap even at the gene family level, except for *small heat shock proteins* (*sHSP*) although from a different *sHSP* family in each tissue (fig. 4). We find that scores of novel genes have evolved in the dung beetle clade, more prominently so in accessory glands than in testes (fig. 1). Among the 45 highest expressed accessory gland genes in *C. molossus*, a total of 69% (31) have evolved in the beetle clade but only 7% (3) are conserved in all three beetle species up to *T. castaneum*. The remaining 62% (28) of genes originated in the dung beetle clade, but 51% (23) evolved specifically in the *C. molossus* genome, whereas only 11% (5) are shared between *O. taurus* and *C. molossus*. Male accessory gland proteins of insects are known to evolve rapidly, not only in terms of sequence divergence but also at gene compositional level (Swanson et al. 2001; Goenaga et al. 2015; Abry et al. 2017; Bayram et al. 2019). Our findings, showing the evolution of striking gene compositional differences in the accessory glands of even closely related dung beetle groups, reflect a similar trend (fig. 1). The two dung beetle species examined, *C. molossus* and *O. taurus*, belong to the same subfamily *Scarabaeinae* and are likely separated from each other by only 20M years, as estimated using the oldest split (Gunter et al. 2016). Such trends of rapid evolution of novel testes and accessory genes have been suggested in several insect groups (Nielsen et al. 2010; McGraw et al. 2015; McDonough et al. 2016; Izquierdo et al. 2019; Sirot 2019), but to our knowledge there has been no detailed comparative investigation across beetles and flies thus far. In our study, although 77% (30) of genes are highly conserved in insects (and in metazoans in general), we see a higher number of testes genes evolving specifically in the dung beetle clade. Of the 33% (15) gene that originated in beetles, 22% (10) have evolved in the dung beetle clade with only 4% (2) found only in the genome of *C. molossus*.

### The Molecular Basis for the Origin and Evolution of Novel Reproductive Genes in Dung Beetles

Fast evolving insect genomes have been the focus of many studies investigating phenotypic trait evolution. Reproductive traits are prime candidates for such studies because rapid evolution of new reproductive genes can lead to rapid reproductive specialization, and this can have important implications for postmating sexual selection and for incipient speciation within a species (Goenaga et al. 2015; Yamane et al. 2015). However, the molecular evolutionary basis of reproductive genes as a complex phenotypic trait has remained understudied in insects. Here, we show that a large number of genes expressed in both the accessory glands and testes are specific to the *C. molossus* genome, and they are not shared even with the closely related *O. taurus* (fig. 2). Further, many genes in the dung beetle clade have no genomic counterparts in *T. castaneum* or *D. melanogaster* (fig. 2). These data, together with evidence from expression of these evolutionary novelties in male reproductive tissues (fig. 4) , indicate that expansion and contraction of dung beetle genomes and evolution of tissue-specific expression patterns could mediate reproductive trait evolution in *C. molossus*. Although this is more apparent in the accessory glands which accounts for more than two dozen novel dung beetle genes, even the testes are subject to similar modes of evolution although to a much lesser extent (fig. 2). The absence of even weak levels of homology to genomic regions in ancestral insects indicates that completely novel genomic scaffolds, specific to *C. molossus* or to the dung beetle clade, may likely be the genetic source for the origin of novel reproductive genes in dung beetles.

Further, we find evidence that the genomic material for one of *C. molossus* accessory gland genes is likely derived from horizontal transfer of genomic material from a fungus, rather than from an ancestral insect. Initial BLASTP in *nr* returned hits to fungal species, and subsequent searches in EggNOG v.5.0 database (Huerta-Cepas et al. 2019) revealed putative orthologous genes in fungi and in other insects. Reconstruction of a Bayesian phylogeny using ortholog groups suggests that genomic DNA from *P. infestans* or an ancestral fungal species could have become incorporated into the genome of *C. molossus* and into the genomes of other insects, including flies, butterflies, and a mosquito species (supplementary fig. S1, Supplementary Material online). Although the *C. molossus* sequence bears overall 30% identity across 91% of *P. infestans* sequence (*e-value* 5e-10), it also contains a secretory signal followed by a *kazal-like* domain that bears an almost equal similarity to the soil fungus *Mortierella* (42% identity over 95% of the sequence; *e-value*** **=** **2-e07; NCBI accession number: KAF9152449.1) and to a related protein called *turripeptide Pal9.2-like* in *O. taurus* (47% identity over 79% of the sequence, *e-value*** **=** **2-e07; NCBI accession number: XP_022919292.1). It is possible that fungal genomic fragments were originally incorporated into insect genomes and may have subsequently undergone divergent evolution in *C. molossus*, *O. taurus*, and other insect species to give rise to completely different proteins.

Other potential mechanisms of novel gene evolution in dung beetles could involve sequence divergence or postgenomic events, such as differential regulation of gene expression for tissue-specific expression. Further investigation of such mechanisms would require concerted data collection efforts targeting more recent divergences i.e., within same genus or even sister taxa.

Given that genes in certain insect and functional categories show specific patterns of high representation and high expression in either the accessory gland or in the testes (figs. 2*c*, 3*c*, and 3*d*), we tested the hypotheses that there could be an association between evolutionary status of gene and their expression levels. Specifically, we were interested in finding whether genes that are newly recruited into *C. molossus* genome are also recruited for high expression in the accessory glands; and whether conserved insect genes are associated with higher expression in the testes. However, we do not find a significant association with gene expression levels in either the testes or accessory glands for samples from both localities (supplementary table S2, Supplementary Material online). Recruitment of a gene for higher or lower expression therefore appears to be a dynamic process, unrelated to the gene’s status as newly recruited younger gene or evolutionarily conserved older gene.

### Functions of Male Reproductive Genes in *C. molossus*

#### Spermatogenesis and Sperm Function Genes in the Testes

Insect spermatogenesis is a complex process orchestrated by a variety of genes, gene products, and long non-coding RNAs, and diverse families of proteins and protein domains, such as *tubulins*, *aminopeptidases*, *dyneins*, *proteases*, and chaperone proteins, all of which have been identified as crucial for sperm production, sperm cytoskeletal structure as well as for sperm function (Vibranovski et al. 2009; Wasbrough et al. 2010; Dorus et al. 2011; Vedelek et al. 2018; Laurinyecz et al. 2019). We find nine annotated genes from six gene families that play important and distinct roles in spermatogenesis and sperm function, and many of these genes are highly expressed exclusively in the testes of *C. molossus* (fig. 2*b and c*). These gene families include *jupiter*, *tubulin*, *dynein light chain*, *leucyl aminopeptidase*, *porin or voltage-dependent anion channel* (*VDAC*), and *deleted in azoospermia* (*DAZ*) (fig. 4*b*).

Among genes with cytoskeletal function is the RNA-binding gene *lark,* which encodes RNA recognition motifs. It is involved in organizing actin cytoskeleton of eggs and is therefore required for fertility of female flies (McNeil et al. 2001, 2004), but a male-specific role for *lark* is still unclear. *Jupiter* belongs to a family of microtubule-associated proteins, and it is thought to perform cytoskeletal functions (Karpova et al. 2006). It is expressed in *C. molossus* testes, and is among the highest expressed genes in the testes of flies although the exact functions are unclear (Fischer et al. 2012). *Jupiter’s* association with and its property of binding to tubulins in flies (Karpova et al. 2006), could be indicative of a role in spermatogenesis because the *tubulin* family produces structural and cytoskeletal proteins that constitute eukaryotic sperm. We find two isoforms of *alpha-tubulin* and one *beta-tubulin* co-expressed in *C. molossus* testes in high to moderate levels (fig. 4*b*). They are structural and cytoskeletal constituents of sperm axonemes and are involved in microtubule based movement of spermatids and spermatocytes (Wasbrough et al. 2010). Both *alpha and beta tubulins* are abundantly expressed proteins in the testes and sperms of *Drosophila*, second only to another family of genes called *leucyl aminopeptidases* which are twice as abundant as *tubulin* proteins (Vibranovski et al. 2009; Wasbrough et al. 2010; Dorus et al. 2011).

Interestingly, we also find two isoforms of *leucyl aminopeptidases* among *C. molossus* testes genes, and both *tubulin and leucyl aminopeptidase* are among the highest expressed annotated gene families in *C. molossus* testes (fig. 4*b*). *Leucyl aminopeptidases* that occur in the sperm are called *Sperm-leucyl aminopeptidases* (or *S-LAPs*), and they are a family of multiple genes that have undergone high levels of diversification and potential neofunctionalization in insects (Dorus et al. 2011). Although their specific functions are still largely unknown, *S-LAPs* are expressed exclusively during spermatogenesis and specifically during the developmental window of spermiogenesis and sperm production and are hence thought to play an important role in sperm function (Wasbrough et al. 2010; Dorus et al. 2011; Laurinyecz et al. 2019).

We find one *porin/VDAC* isoform moderately expressed in the testes *C. molossus*. *Porins* are mitochondrial transmembrane pore-forming proteins that facilitate mitochondrial energetics and diffusion of metabolites, and they have been found to be abundantly expressed in both male and female germ cells of flies (Guarino et al. 2006; Specchia et al. 2008). *Porin 2* is highly expressed specifically in testes and is localized to the tail of spermatozoa where mitochondria and cytoskeletal structures involved in cell movement occur (Guarino et al. 2006; Specchia et al. 2008). *Porins* are critical for male fertility in *D. melanogaster* as disruption of *Porin* loci leads to male sterility (Oliva et al. 2002).

Also, critical for animal spermatogenesis is the *DAZ* gene family, and as the name “*Deleted inAzoospermia*” suggests, absence or abnormalities of genes in this family result in deficiencies or lack of sperm production. The *DAZ* family consists of *Boule (bol)* conserved in metazoans, *DAZ-like* (*DAZL*) found in vertebrates, and the *DAZ* gene found only in primates. We find one moderately expressed transcript from the *DAZ* family in the testes of *C. molossus* which is likely related to or derived from *bol* (fig. 4*b*), because *bol* gene products have been found in several insect orders including in coleoptera and it is the only gene member of the *DAZ* family found in *Drosophila* (Cheng et al. 1998; Adams et al. 2000; Honeybee Genome Sequencing Consortium 2006; Tribolium Genome Sequencing Consortium 2008; Werren et al. 2010; Sekine et al. 2015). *Bol* gene isoforms have been shown to be essential for sperm maturation and differentiation during meiotic processes of spermatogenesis in insects (Cheng et al. 1998; Sekine et al. 2015). *DAZ* isoforms contain one or more repeats of a characteristic 24 amino acid RNA-binding motif called *DAZ* repeats that bind to specific mRNA targets and control target mRNA transport and translation in the cytoplasm (Fu et al. 2015).

Among other genes involved in spermatogenesis and sperm function is the *dynein light chain (Dlc)* family. *Dlc* consists of multifunctional genes that participate in cytoskeletal and cellular events required for sperm elongation, maturation, individualization, and are key to the formation of active sperms in insects (Ghosh-Roy et al. 2005). We identified one isoform of *Dlc T-complex testis-specific type* (*Dlc Tctex-type*) moderately expressed in *C. molossus* testes, and it is 70% identical to a 111-residue protein *Dlc90F* in *D. melanogaster. Dlc90F* is essential for spermatid differentiation and forms part of a cytoplasmic dynein complex which links to cellular targets that regulate *dynein* function (Li et al. 2004). *Dlc* perform similar functions in oocytes, where they form a cytoplasmic microtubule motor complex (*dynein motor complex*) that interacts with other proteins to transport mRNA and proteins to their site of action (Navarro et al. 2004). *Dlc’s* have also been implicated in other important insect cell functions such as protein clearance, autophagy, and cell death (Batlevi et al. 2010).

#### Stress Response and Immunity Genes

*Heat shock proteins (HSPs)* are an important and diverse family of proteins that drive cellular stress response and control cell damage. Among these, are a class of small 12–43 kDa proteins called *smallHSPs (sHSPs)* which are ubiquitous, broadly expressed, and found in multiple cell types (Haslbeck 2002; Haslbeck et al. 2005; Basha et al. 2012; King and MacRae 2015). Seven isoforms of *sHSP20*, also called *HSPB6*, are expressed in moderate to low amounts in the accessory glands of *C. molossus*. *sHSPs* are ATP-independent and perform a plethora of functions including facilitating clearance of aggregated non-native proteins and metabolites through protein-binding and chaperone mechanisms (Bukach et al. 2004; Basha et al. 2012). In addition to this important role as molecular chaperones, *sHSPs* inhibit apoptosis, organize and remodel cytoskeleton, and serve as structural proteins in the sperm (Haslbeck 2002; Franck et al. 2004; Basha et al. 2012; King and MacRae 2015). In humans, *sHSP20* is broadly expressed in many tissues, acting as co-chaperones to prevent platelet aggregation and also involved in relaxation of smooth muscles (Bukach et al. 2004; Basha et al. 2012). In insects, *sHSP20* is important for cell survival during temperature change and regulate diapause (Gkouvitsas et al. 2008).

*14-3-3 Epsilon* is among the highest expressed genes in *C. molossus* testes (fig. 4*b*). The *14-3-3* family consists of phosphoserine/phosphothreonine-binding proteins that interact with hundreds of other proteins and are involved in the regulation of subcellular localization, protein-protein interactions, cell cycle, intracellular signaling, and apoptosis (Morrison 2009). One of the protein families that interacts with *14-3-3 Epsilon* is *akirin*, and we find one *akirin* isoform moderately co-expressed in *C. molossus* testes. *Akirins* are nuclear factors with important roles in innate immune response, regulation of gene transcription, and are crucial to embryonic development as knockdown causes lethal embryonic phenotype (Goto et al. 2008; Macqueen and Johnston 2009; Chen et al. 2013; Bonnay et al. 2014). *Akirins* lack binding motifs, so instead of directly binding to nucleic acids they interact with cofactors and transcription factors such as *14-3-3 Epsilon and NF-kappaB*, to regulate mRNA transcription and immune responses (Chae et al. 2003; Goto et al. 2008).

Additionally, *14-3-3 Epsilon* is the binding partner and molecular chaperone of *HSPs*, specifically *HSP60*, and it is widely expressed in multiple tissues of insects including in the testes (Tabunoki et al. 2008). *HSP60* or other larger *HSPs* are not among the highest expressed genes examined in either testes or accessory glands of *C. molossus*. However, in both tissues we find *sHSP* families expressed in low to moderate levels, with one *HSP10* expressed in the testes and seven isoforms of *HSP20* in the accessory glands (fig. 4). Insect *sHSP’s*, including *HSP10 and HSP20*, are involved in stress response, immune response, protein folding, and they act as cochaperones of larger *HSP’s* (Sadacharan et al. 2001; Gorshkov et al. 2015; King and MacRae 2015). It is likely that *sHSP’s* expressed in the testes and/or accessory glands of *C. molossus* could be acting as cochaperone proteins and/or interacting with other testes or accessory gland proteins.

We also find a suite of other *C. molossus* testes genes with potential roles in stress and immune response, including *coenzyme Q (CoQ)*, *glyceraldehyde-phosphate dehydrogenase (GADPH)*, *nudix*, *periredoxins and thioredoxins*, *Bax inhibitor-1 (BI-1)*, and *coiled coil-helix-coiled coil-helix (CHCHD)*. In insects enzymes such as *CoQ* and *GADPH*, which were traditionally considered to be crucial metabolism and energy pathway genes, have recently been found to perform alternate functions in stress response and defense. *GAPDH* is now known to perform multiple roles in apoptosis, neuronal transcription, export of nuclear RNA, and in DNA repair (Chuang et al. 2005). *CoQ*, a well-established component of oxidative phosphorylation in the mitochondria for ATP synthesis is now known to be an essential antioxidant that inhibits cell death (Crane 2001). *Nudix* genes are also enzymes, and they have evolved variable number of *nudix* domains through gene duplication/fusion events (Lin et al. 2009). Several isoforms of *nudix* are specifically expressed in the mammalian testis, and they interact with testis-specific *sHSPs*, eliminating toxic nucleotide derivatives and regulating the levels of important signaling nucleotides and their metabolites (McLennan 2006; Lin et al. 2009).

Among other stress and immune response genes are ubiquitous scavenging enzymes *periredoxins and thioredoxins* which are well established as stress response gene families that protect cells and cell organelles from oxidative stress. *Periredoxins* remove peroxides and peroxynitrates from cells to prevent cell damage and maintain cellular homeostasis (Perkins et al. 2015; Rhee 2016). *Thioredoxin* domain containing proteins are important for immune response and defence against oxidative stress-induced cell death (Thulasitha et al. 2016). *Bax inhibitor-1 (BI-1)* is an evolutionarily conserved protein involved in apoptosis cascades in the mitochondria and stress response in the endoplasmic reticulum (Lisbona et al. 2009). *BI-1* is known to have cryoprotective functions and inhibits cell death induced by oxidative stress and heat shock (Chae et al. 2003). *CHCHD* proteins are predicted to have redox properties and to perform oxidoreductase and/or metallochaperone functions (Banci et al. 2009).

#### Regulation of Protein Synthesis

The testes of *C. molossus* express multiple isoforms of *eukaryotic initiation factors (eIF)* which are key players in various stages of initiation of mRNA translation during eukartyotic protein synthesis. The ultimate function of *eIF* is to regulate translation initiation by recruiting and regulating *ribosomal RNA subunits 40S and 60S* and forming translation initiation complexes with each other as well as with non-*eIF* proteins (Pelletier and Sonenberg 2019). We find five isoforms including *eIF1*, *eIF1A*, *eIF4E1/eIF4E*, and *eIF4H*, with high to moderate expression in *C. molossus* testes. Of these, *eIF1 and eIF1A* are inspectors of translation initiation codons (AUG), and they mediate the formation of *40S* preinitiation complex to regulate translation (Chaudhuri et al. 1997; et al. 1999; Miyasaka et al. 2010; ). We find two isoforms of *eIF4E1/eIF4E*, a 5′ mRNA cap-binding protein which is the rate-limiting protein that regulates/represses/silences translation initiation (Kinkelin et al. 2012; Pelletier and Sonenberg 2019). *eIF4E* has many binding targets including *cup* which represses the translation of embryogenesis genes in female flies (Kinkelin et al. 2012)*.* We also find *eIF4H*, a broadly expressed protein which works in conjunction with and forms complexes with other *eIF’s* to unwind and prevent reannealing of 5′ UTR mRNA and facilitate binding to *40S ribosomal subunit* (Rogers et al. 2001; Pelletier and Sonenberg 2019).

*Nucleosome assembly protein 1* (*NAP-1*) is a histone chaperone and forms an integral component of eukaryotic chromatin (Park and Luger 2006). Investigations of *NAP-1* crystal structure and functions in *D. melanogaster* revealed it is involved in chromatin remodeling, telomere stability, and transcription, as well as transcriptional regulation of many genes through histone shuttling into nucleus and assembly of nucleosomes (Park and Luger 2006; Lopez-Panades and Casacuberta 2016).

#### *Female*, *Egg*, *or Embryo-Related Genes in the Accessory Glands*

The accessory glands express two females, eggs, or embryo related genes. The *general odorant-binding protein 56d (Obp56d)* is involved in olfactory and pheromone perception, and it is expressed in fly antennae (Larter et al. 2016; Campanini et al. 2017). Reproductive stage expression differences have also been noted in male fly antennae (Campanini et al. 2017). However, expression of Obp56d in the accessory glands of beetles has not been previously documented and the functions remain to be investigated. The *fascilin* family of membrane glycoproteins are from the *immunoglobulin* superfamily, and they are developmental proteins that play a role in cell adhesion, differentiation, and neurogenesis in insect embryos (Zinn et al. 1988; Grenningloh et al. 1991). *Fascilin II* is secreted in the male accessory glands of only mated lepidoptera, and it is thought to have a function in oogenesis (Saraswathi et al. 2020). *Fascilins* are found in embryogenic tissues of flies and grass hoppers and are hence thought to be egg and embryo-related insect proteins (Zinn et al. 1988; Grenningloh et al. 1991).

### Structure-Function Analysis of Novel Genes with Unknown Function

Functional annotation of several genes in both the testes and accessory glands is not possible through primary protein sequence alignments because they are completely novel to *C. molossus* and do not return BLAST hits to any other living organism in the *nr* database. Therefore, functional annotation based solely on alignment of sequence for these novel proteins is not feasible. Different amino acid sequences may fold into similar structures (Krissinel 2007), and similarity in three-dimensional structures potentially imply similarity in function. Therefore, searches using predicted structures for structurally similar hits should further assist functional annotation of these novel proteins. Our results from I-TASSER suite (Yang et al. 2015) suggest that most of the accessary gland novel proteins are rich in helical structures, with structural homology to domains within typically larger complexes of transmembrane transporters (e.g., C126_AG_Sequence_13 and C126_AG_Sequence_15) or bacterial secretion systems (e.g., C126_AG_Sequence_7 and C126_AG_Sequence_15) that are involved in ion or protein transport, suggesting possible structural roles for these highly expressed proteins (supplementary tables S3 and S5, Supplementary Material online). Similarly, helix-rich structures of the testes proteins have structural homology to domains of proteins in bacterial secretion systems (e.g., C126_T_Sequence_20) or structural proteins with motor transport function (e.g., C126_T_Sequence_2) (supplementary tables S4 and S5, Supplementary Material online). Among all three-dimensional structure–function analyses, TM align structural alignments returned highest confidence results, with TM scores ranging from 0.606 to 0.79 for accessory glands and 0.524 and 0.961 for testes (supplementary table S5, Supplementary Material online). TM align performs structural alignment of the first I-TASSER predicted model to all structures in the PDB library to check overall similarity in the protein folds. Hits to the targets in PDB library with the highest TM score can therefore be used to infer structural and hence functional similarity of query protein. We find two proteins in the accessory glands that are structurally similar to *exportin-4* (TM score 0.738) and *V-type proton ATPase subunit G* (TM score 0.606) and could hence be involved in transmembrane and nuclear transport of proteins/ions. A third accessory gland protein with similarity to *von Willebrand factor* (TM score 0.612) may perform binding/chaperone functions and maintenance of homeostasis (supplementary table S5, Supplementary Material online). In the testes, we find proteins similar to *WD repeat-containing protein 34* (TM score 0.879) with dynein-binding functions involved in cilium assembly and microtubule-based and interciliary transport. Interestingly, we find *dynein light chain* among the annotated spermatogenesis and sperm function genes in the testes of the *C. molossus*. Testes proteins also showed matches to *anosmin-1* (TM score 0.961) and *tripeptidyl-peptidase 2* (TM score 0.524) both of which act as proteases, peptidases and protease inhibitors. Although both tissues have two proteins each with hits in the PDB library, the functions of these cannot be interpreted as the target proteins functions have not yet been elucidated.

On the whole, our predictions based on ligand binding sites returned low scores. Therefore, instead of pin-pointing particular functions based on these predictions, we find that considering the consensus GO terms associated with the best aligned structure could be more reliable (supplementary table S6, Supplementary Material online). The highest expressed proteins in the accessory glands returned predicted molecular functions that include nucleic acid-binding transcription factor activity; pyrophosphatase activity; purine ribonucleoside triphosphate binding; guanyl ribonucleotide binding; signal transducer activity, substrate-specific transporter activity biological process: intracellular transport; cellular protein localization; establishment of protein localization, and endopeptidase inhibitor activity (supplementary table S6, Supplementary Material online). These functions indicate that the proteins could be involved in biological processes, such as regulation of gene expression; regulation of cellular macromolecule biosynthetic process; regulation of RNA metabolic process, taxis, signal transduction; intracellular transport; cellular protein localization; establishment of protein localization, negative regulation of hydrolase activity and regulation of peptidase activity (supplementary table S6, Supplementary Material online). The predicted molecular functions of the testes included transport; nucleic acid and protein binding; and peptidase inhibitor and regulatory activities; and they are involved in biological processes such as RNA transport and regulation of RNA metabolic process; cellular and intracellular transport; protein localization; nucleosome assembly; regulation of gene expression; regulation of cellular macromolecule biosynthetic process. However, the results from these GO terms also need to be interpreted with caution given the limitations that the highest overall Cscore^GO^ is 0.50 for accessory glands and 0.61 for the testes (on a scale of 0–1) and also due to the layered nature of consensus prediction.

## Conclusions

Insects have been the focus of many studies on phenotypic trait evolution because their genomes are fast evolving. Although male ejaculates and male reproductive proteins in insects represent a complex and highly variable phenotypic trait, little is known about the molecular basis of reproductive trait evolution. Here, we examined the male reproductive genetic profiles and male reproductive gene evolution in the dung beetle *C. molossus* using de novo transcriptome sequencing of accessory glands and testes. We show that novel reproductive traits evolve through rapid expansion and contraction of genomes and gene repertoires in dung beetles. Scores of *C. molossus* reproductive genes are novel and are not shared even with closely related dung beetles. The accessory glands are particularly important for the evolution and diversification of novel secretory genes, several of which become recruited for high expression. The testes of *C. molossus* are primarily orientated toward evolutionarily conserved non-secretory genes, several of which are implicated in spermatogenesis and sperm function. There is, however, no association between the evolutionary novelty/conservation status and gene expression levels. We also find that the accessory glands and testes are largely functionally highly specialized to the gene level, although a few broad functions such as stress response, immunity, and protein biosynthesis functions are shared. Further, structural analysis of novel genes of unknown function suggests bacterial secretion systems, with accessory gland genes potentially involved in ion or protein transport and testes genes associated with motor transport functions. This study sheds light on the molecular basis of evolution of novel male primary reproductive traits, which represent complex phenotypes. Specifically, our findings of rapid evolution of accessory gland genes support the prediction that seminal fluid proteins involved in postcopulatory reproductive success may be under strong divergent selection. This study provides a foundation for future investigations into molecular mechanisms mediating postcopulatory male-female interactions in dung beetles.

## Materials and Methods

### Field Work and Sample Collection

*Catharsius molossus* is endemic to Singapore and South East Asia (Ong et al. 2013). Fieldwork was conducted to collect dung beetles using dung-baited pitfall traps (National Parks Board sampling permit: NP/RP18-034-1b) from two localities: Pulau Ubin, an island in the Straits of Johor located to the north east of Singapore mainland (1º24′49.9″N; 103º58′36.0″E) on February 20, 2019; and Upper Peirce, located in the central part of mainland Singapore (1º22′31.8″ N; 103º46′51.8″E) on April 30, 2019. Live *C. molossus* male specimens were transported back to the laboratory and were treated at −80ºC for 10 min prior to dissection. One major male beetle was chosen from each site, and for each beetle, two tissues were collected separately: testes and accessory glands. The four dissected tissue samples were immediately snap frozen on dry ice to preserve RNA integrity.

### Total RNA Isolation

Immediately after dissections were complete, 400 µl RNAzol RT was added to each tube and the tissue was pulverized using a polytron pestle and handheld homogenizer. Another 600 µl of RNAzol RT was added and mixed to the homogenate, and the tubes were stored at −80ºC until extraction, at which point the sample tubes were thawed on ice. DNA, proteins, and polysaccharides were first precipitated by adding 400 µl of nuclease-free water followed by vigorous shaking for 15 s. The samples were allowed to stand for 15 min at room temperature and then centrifuged at 12,000 × g for 15 min at 4ºC. To precipitate the RNA, 750 µl of the supernatant was transferred to a new tube and 750 µl of 100% isopropanol was added, followed by a brief vortex and incubation at 10 min at room temperature. The samples were centrifuged at 12,000 × g for 10 min at room temperature. The supernatant was discarded, and the RNA pellet was washed twice with 500 µl of 75% ethanol with centrifugation at 8,000 × g for 3 min at room temperature. The final ethanol wash was discarded, and any residual ethanol was removed with a micropipette taking care not to dislodge the pellet. The extracted RNA was resuspended in 22 µl nuclease-free water and vortexed at room temperature for 5 min. A total of 2 µl of extract was used for preliminary RNA quality checks and quantification on a Qubit, and the remaining sample was stored at −80ºC.

### Transcriptome Sequencing

The four RNA samples were transferred on dry ice to NovogeneAIT Genomics Singapore Pte Ltd for cDNA library construction and RNA sequencing (RNAseq). A bead-based poly-AAA tail selection was used to enrich samples for mRNA. NEBNext UltraTM Directional Library Prep Kit was used for cDNA libraries construction and quality checks of the four libraries were done on Agilent 2100 Bioanalyzer. A total of 250 bp paired-end (PE) sequences were generated on Illumina NovaSeq 6000 platform.

### Raw Read Cleanup

Dung beetles are hosts to several types of nematodes, bacteria, and viruses, and some of these are especially prevalent in reproductive tissues. Moreover, dung beetles are coprophagous and mammalian contamination is also possible. Hence, we first cleaned the raw reads for contaminant sequences by searching the reads against worm, bacterial, viral, and human sequence databases. For worm decontamination, we created a custom database by downloading 2 million worm mRNA sequences from NCBI. Sequences were dereplicated using PRINSEQ v0.20.4, and all sequences ≥ 200 bp without too many ambiguous base pairs were retained. The filtered worm mRNA sequences were converted into a worm database using DECONSEQ v0.4.3 (Schmieder and Edwards 2011a, 2011b). For bacterial, viral, and human contamination, readymade databases available through DeconSEQDB were used. The four dung beetle transcriptomes were searched against all four databases and a cut-off of 95% sequence identity and 90% alignment coverage was used to call contaminants.

### De Novo Transcriptome Assembly

Cleaned reads were processed in Trimmomatic-0.32 to trim adapters and primers (Bolger et al. 2014). A sliding window Phred score of Q20 was applied across 4 bp for quality trimming base pairs and all reads ≥100 bp were retained. The quality trimmed reads were used for de novo transcriptome assembly in Trinity v2.8.6 (Grabherr et al. 2011). For each sample, both accessory gland and testes reads were assembled together. The de novo assembled sequences were translated in six frames, and ORFs were predicted de novo in OrfPredictor v3.0 (http://bioinformatics.ysu.edu/tools/OrfPredictor.html; last accessed August 11, 2021) (Min et al. 2005). Duplicate sequences with 100% identity at the nucleotide level were removed using CDHIT v4.7 (Weizhong and Godzik 2006; Fu et al. 2012). The final filtered assembly consisted of all sequences ≥200 bp in length.

### Curation of De Novo Assembled Sequences

*Catharsiusmolossus* is a non-model dung beetle species, and genomic resources for dung beetles in general are still scarce. Only one other dung beetle *O.taurus*, which is from the same subfamily *Scarabaeinae* has a genome and gene level annotations that are publicly available. To mitigate the lack of appropriate genic databases for *C. molossus* and to identify and remove chimeras or any remaining contaminant sequences, we performed extensive manual curation and filtering of the de novo transcriptome assemblies of *C. molossus*. We first applied a gene expression-based cut-off by mapping the quality-controlled reads back to the transcriptome assemblies using SALMON v1.0.0 (Patro et al. 2017). We selected the top 100 highest expressed genes in each tissue for each sample taking into account the steep drop in gene expression beyond this number, especially in the accessory glands. We then curated the 400 sequences for assembly accuracy by searching against, *O. taurus*, *T. castaneum*, and *D. melanogaster* databases using BLASTX to identify insect genes and then by BLASTP against the complete *nr* database in NCBI. Any sequences that represented chimeras, bacterial contaminants, or rRNA were removed. Finally, within each tissue, sequences were dereplicated at 100% protein identity using CD-HIT v4.7 (Weizhong and Godzik 2006, Fu et al. 2012). The final number of high-quality sequences retained for downstream analyses was 45 per sample per tissue.

### Evolutionary Analysis of Filtered Gene Set

For the purpose of this study, we define rapid evolution as the rate of acquisition and loss of de novo protein-coding genes by a species, leading to the evolution of functional diversity. We used the filtered sequences (45 per sample per tissue) for downstream evolutionary and functional analyses. To evaluate loss and gain of reproductive genes in *C. molossus*, we interrogated our sequences against three additional insect species: *O. taurus*, *T. castaneum*, and *D.melanogaster*. Genomic databases for these species as well as all available sequences on NCBI (protein and nucleotide) were examined. The genomic assemblies used in this study are: Otau_2.0 (accession number: GCF_000648695.2) submitted by The i5k Initiative (Goldsmith et al. 2013); *T. castaneum* Tcas5.2 (accession number: GCF_000002335.3) (Richards et al. 2008; Kim et al. 2010) and *D. melanogaster* Release 632 (accession number: GCA_000001215.4) (Adams et al. 2000; Hoskins et al 2015).

In addition to availability of genomic resources, these three species were selected as evolutionary reference points for the following reasons: *O. taurus*, a dung beetle belonging to the same subfamily, is a close relative of *C. molossus* (Gunter et al. 2016)*; T. castaneum* is a well-studied model species belonging to the same order; and finally, *D. melanogaster* provides a more distant evolutionary reference point and as a model species among insects, offers a well-annotated genome and gene set that is largely functionally characterized. Using the curated set of the top 45 highest expressed, high-quality genes from each tissue of male *C. molossus*, BLAST searches were performed against the protein databases each of these three species. Any genes found to be absent in the protein database were further searched with BLASTN, BLASTX, TBLASTN, and TBLASTX against entire genomes of other insect species to rule out the possibility of calling gene absences due to incorrect/undocumented gene models or due to coding genes arising from ancestral non-coding genomic regions. An *e-value* cut-off of 1*e*-5 was used and gene presence and absence were called based on BLAST results and evaluation of alignments to BLAST hits. In cases where exact gene assignment was confounded by presence of multiple isoforms in large gene families or by a high rate of sequence evolution, we considered the putative gene to be shared among species pairs. This is a more conservative approach as opposed to assigning the gene as novel to *C. molossus*.

### Analysis of Association between Evolutionary Status and Gene Expression

We tested the hypothesis that novel accessory gland genes in the *C. molossus* genome may become recruited for high gene expression as opposed to gene sets that are conserved in other insect species (in dung beetles, in beetles, or in all four insects). Conversely, in the testes, we tested whether high expression tends to be associated with genes that are evolutionarily conserved. Based on whether gene expression was above or below the median expression value within each sample, we classified each gene as high or low expressed. We then performed a *Chi-squared* test in PSPP 1.4.0 (Stover 2010; Pfaff et al. 2011) to determine whether there is an association between gene expression level and gene conservation.

### Analysis of Potential Horizontal Gene Transfer into *C. molossus* Genome

One of the accessory gland genes that was specific to *C. molossus*, but had significant BLASTP hit (*e-value* 1*e*-5) to fungi in NCBI’s *nr* was further investigated for HGT which is common in insect genomes. The protein sequence of *C. molossus* was used to search for orthologs in EggNOG v.5.0 database (Huerta-Cepas et al. 2019), and all ortholog groups that returned a hit were downloaded. Sequences were checked for duplication at the protein level using CDHIT v4.7 (Weizhong and Godzik 2006; Fu et al. 2012) and then filtered based on BLASTP *e-value* cut-off of 1*e*-5 and clustering with *C. molossus* sequence. Filtered sequences were aligned using MUSCLE and trimmed in MEGA X version 10.2.6 (Kumar et al. 2018; Stecher et al. 2020). Mixed-model Bayesian analysis in amino acid setting was implemented in MrBayes 3.1 (Ronquist and Huelsenbeck 2003). Four independent MCMC analyses of 10 M cycles each (sampled every 1,000 generations) were performed with four chains. The first 25% of trees were discarded as burn-in and a 50% majority-rule consensus tree was constructed from combined post-burn-in trees.

### Functional Analysis of Filtered Gene Set: Gene Annotation, Secretory Nature, and Gene Expression

Functional annotations of the *C. molossus* genes were assigned based on BLAST searches. For each tissue, homologous pairs of sequences between Pulau Ubin and mainland Singapore samples were resolved based on reciprocal BLASTP. A final gene expression quantification was performed by remapping the filtered reads against the high-quality gene set to calculate TPM using SALMON v1.0.0. The TPMs were log_2_ transformed and scaled before plotting as a heatmap using *pheatmap* function in *R* (RStudio 1.1.453). Finally, coding DNA sequences of all 45 genes were translated into protein sequence in MEGA7, and SignalP 5.0 webserver was used to check for the presence of secretory signals, a feature particularly important to glandular proteins.

### Structure and Function Predictions of Novel Accessory Gland Proteins

In order to better understand the functions of novel accessory gland genes, the top five highest expressed genes were selected for analysis. Translated amino acid sequences of these genes did not show significant similarity in primary structure with proteins of known function when searched against UniProt database (UniProt Consortium 2019). Further protein secondary and tertiary structure and function prediction were performed through algorithms as implemented by the I-TASSER suite (Yang et al. 2015). SignalP v. 5.0 server (Almagro Armenteros et al. 2019) was used to remove signal peptides from the protein sequences and trimmed sequences submitted for prediction. The sequences were first fed into the Iterative Threading ASSEmbly Refinement (I-TASSER) server for three-dimensional structure modeling by iterative threading assembly simulations. Next, the structure model with the best confidence score were aligned to all available structures in the Protein Data Bank (PDB) using the TM-align algorithm. For further inferences of possible functions, the predicted models were fed into COFACTOR and COACH algorithms to match for potential ligand-binding sites, enzyme active sites, and consensus gene ontology terms (GO terms) based on structure comparisons and protein-protein networks analysis.

## Supplementary Material

evab172_Supplementary_Data

## Data Availability

The data underlying this article are available in GenBank at https://www.ncbi.nlm.nih.gov/ and can be accessed with Project ID PRJNA707318.
