# Supplementary material for: Rapid Genomic Evolution Drives the Diversification of Male Reproductive Genes in Dung Beetles
Source: Genome Biol Evol. 2021 Jul 28;13(8):evab172. doi: 10.1093/gbe/evab172 (PMC8382682; doi:10.1093/gbe/evab172)
Supplement: evab172_Supplementary_Data [file evab172_supplementary_data.zip › Fig S1.pdf]

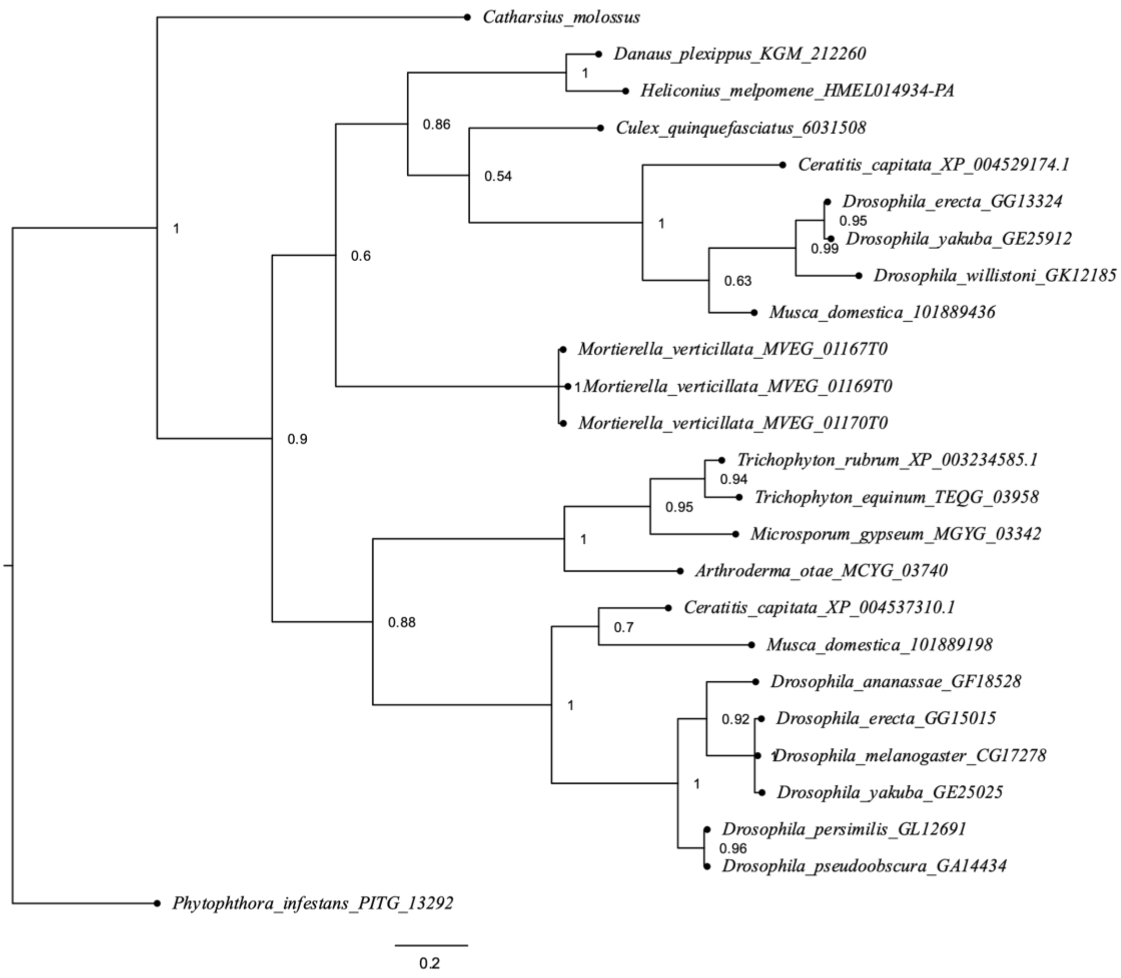

Figure S1. Mixed-model Bayesian phylogenetic reconstruction of a *C. molossus* accessory gland gene with orthologous groups in insect and fungal species (downloaded in EggNOG v. 5.0). Bayesian posterior probabilities are shown in a 50% majority-rule consensus phylogram. Clustering of insect and fungal sequences suggest likely horizontal gene transfer from *P. infestans* or another ancestral fungal species into the genome of *C. molossus* and other insects.
