# Supplementary figures and images for: Rapid Genomic Evolution Drives the Diversification of Male Reproductive Genes in Dung Beetles
Source: Genome Biol Evol. 2021 Jul 28;13(8):evab172. doi: 10.1093/gbe/evab172 (PMC8382682; doi:10.1093/gbe/evab172)

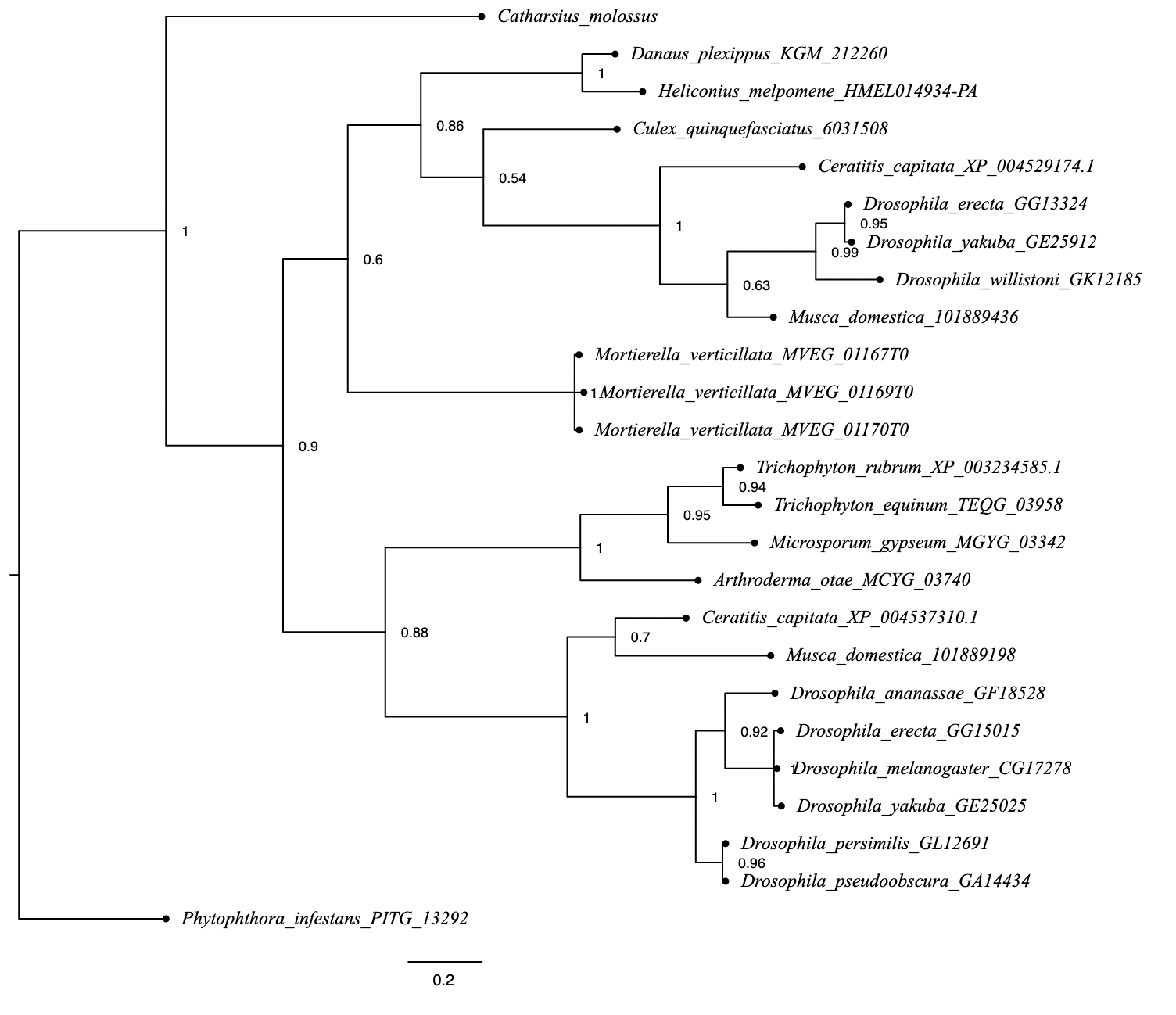

Supplement: evab172_Supplementary_Data [file evab172_supplementary_data.zip › Fig S1. Bayesian Phylogeny.tiff]
